# Preface to the special issue of Phase Separation: methodologies tailored for studying biomolecular condensates

**DOI:** 10.52601/bpr.2022.210901

**Published:** 2022-02-28

**Authors:** Pilong Li

**Affiliations:** 1 Beijing Advanced Innovation Center for Structural Biology & Frontier Research Center for Biological Structure, School of Life Sciences, Tsinghua University, Beijing 100084, China; 2 Tsinghua-Peking Center for Life Sciences, Beijing 100084, China

Cells are super-complex systems that are held far away from equilibrium. One hallmark of the off-equilibrium state is the presence and constant maintenance of subcellular compartments for efficient control of biochemical reactions, material exchange, and information flow. Eukaryotic cells have evolved at least two major ways to compartmentalize their cellular spaces, one aided by enclosure within phospholipid bilayers and the other without. The latter compartments are called membraneless organelles, also named biomolecular condensates. About 13 years ago, it was demonstrated by Hyman, Brangwynne and colleagues that membraneless organelles are formed, at least in part, via a physiochemical process called liquid–liquid phase separation. Soon after that, Rosen and colleagues and McKnight and colleagues demonstrated *in vitro* reconstitution of liquid–liquid phase separation and liquid–solid phase separation, respectively, using simplified biochemical systems. These landmark studies, among others, prompted the start of the field of biomolecular condensates.

Great minds think similarly: scientists in China have been extensively studying all aspects of phase separation and membraneless organelles from the dawn of the field. The 2021 Conference on Biomolecular Phase Separation and Transition (April 8–11, 2021, Southern University of Science and Technology, Shenzhen, China) witnessed the fast and healthy development of the field of biomolecular condensates in China. A large body of excellent national scientists, young and established, with expertise in various areas of biological sciences and related subjects, are actively conducting dynamic research surrounding biomolecular phase separation. The number of conference participants reached the set limit, 500. In total, there were over 50 invited talks covering a wide scope of topics, such as the roles of biomolecular phase separation in neuronal synapsis, in autophagy, in nucleolus organization, in plant flowering control, and in host defense against pathogens.

While this special issue is never meant to comprehensively cover the major techniques utilized for studying biomolecular condensates, it nevertheless collects together some major advances in a number of representative methodologies. Topics in this special issue include: various fluorescence auto- and cross-correlation spectroscopy methods for *in vitro* and *in vivo* characterization of phase separation, the DNA curtains technique for studying phase separation of chromatin regulators and transcription factors, a battery of nuclear magnetic resonance techniques tailored for detection of signals in condensates, technologies for measuring mechanical properties like elasticity and viscosity of condensates, and theoretical modeling of condensation processes. It did not escape the editor’s attention that the physical states of the condensed phases actually cover a continuum from dynamic liquids to solids such as amyloid fibers. Therefore, this special issue also includes two articles concerning the study of solid condensates: one summarizes various *in vitro* biophysical methods for characterizing amyloid fibers, and the other introduces model biological systems for studying amyloid fibers. It is hoped that this special issue will be of great use to researchers studying biomolecular phase separation.

## Conflict of interest

Pilong Li declare that they have no conflict of interest.

